# Validation of Genotyping by Sequencing Using Transcriptomics for Diversity and Application of Genomic Selection in Tetraploid Potato

**DOI:** 10.3389/fpls.2019.00670

**Published:** 2019-05-29

**Authors:** B. M. Caruana, L. W. Pembleton, F. Constable, B. Rodoni, A. T. Slater, N. O. I. Cogan

**Affiliations:** ^1^ Agriculture Victoria Research, Agriculture Victoria, AgriBio, The Centre for AgriBioscience, Bundoora, VIC, Australia; ^2^ School of Applied Systems Biology, La Trobe University, Bundoora, VIC, Australia

**Keywords:** *Solanum tuberosum*, autotetraploid, SNP annotation, variant discovery, GATK, SnpEff, dry matter, crisp score

## Abstract

Potato is an important food crop due to its increasing consumption, and as a result, there is demand for varieties with improved production. However, the current status of breeding for improved varieties is a long process which relies heavily on phenotypic evaluation and dated molecular techniques and has little emphasis on modern genotyping approaches. Evaluation and selection before a cultivar is commercialized typically takes 10–15 years. Molecular markers have been developed for disease and pest resistance, resulting in initial marker-assisted selection in breeding. This study has evaluated and implemented a high-throughput transcriptome sequencing method for dense marker discovery in potato for the application of genomic selection. An Australian relevant collection of commercial cultivars was selected, and identification and distribution of high quality SNPs were examined using standard bioinformatic pipelines and a custom approach for the prediction of allelic dosage. As a result, a large number of SNP markers were identified and filtered to generate a high-quality subset that was then combined with historic phenotypic data to assess the approach for genomic selection. Genomic selection potential was predicted for highly heritable traits and the approach demonstrated advantages over the previously used technologies in terms of markers identified as well as costs incurred. The high-quality SNP list also provided acceptable genome coverage which demonstrates its applicability for much larger future studies. This SNP list was also annotated to provide an indication of function and will serve as a resource for the community in future studies. Genome wide marker tools will provide significant benefits for potato breeding efforts and the application of genomic selection will greatly enhance genetic progress.

## Introduction

Most breeding methods rely heavily on phenotypic selection for germplasm improvement with little importance on genotypic selection at the molecular level. Conventional breeding aims to combine desirable traits together from elite individuals in a step wise manner that is both laborious and time consuming. The resulting time frame can be 10–15 years before the commercial release of a new cultivar (Bradshaw and Mackay, 1994; Jansky, 2009; Slater et al., 2014; Ramakrishnan et al., 2015). Marker-assisted selection (MAS) has been incorporated into the potato breeding cycle for several traits which has resulted in selection of individuals a few years earlier when compared to traditional phenotypic selection and is cost effective (Slater et al., 2013). There are only a few examples of potato breeding programs utilizing MAS, particularly markers linked to disease resistance traits (Ortega and Lopez-Vizcon, 2012; Schultz et al., 2012) and gained efficiencies when compared to phenotypic selection alone. Other more complex traits like most tuber and plant morphological traits, cooking characteristics and yield can be selected for under this scheme (van Eck, 2007; Ramakrishnan et al., 2015). However, due to their polygenic nature, it is more difficult and extremely slow (Hospital, 2009; Slater, 2013).

Genomic selection (GS) predicts the performance of individuals that have been genotyped based on a prediction equation that is derived from an extensively phenotyped and genotyped reference population (Meuwissen et al., 2001). Recent advances in genotyping-by-sequencing (GBS) methods using next generation sequencing (NGS) technologies have been able to deliver the necessary number of genome wide variants for GS at an ever-reducing cost (Davey et al., 2011). This approach is now extensively applied in livestock (Pryce and Daetwyler, 2012; Wolc et al., 2015; García-Ruiz et al., 2016) as well as increasingly in plant breeding (Lin et al., 2014; Crossa et al., 2017). GS requires marker saturation across the genome and aims to estimate the effect of all markers assuming causative mutations are in linkage disequilibrium (LD) with at least one genetic marker. Due to the marker saturation, GS can predict phenotypes for traits under complex polygenic control as well as traits under simple genetic control. Phenotypic selection in conventional breeding programs, particularly for complex traits with high environmental variation and long selection cycles limits genetic gain. However, the application of GS offers the potential to dramatically reduce the generation cycle and increase the rate of genetic gain (Meuwissen et al., 2001). The potential of GS is now being applied to facilitate potato varietal development, and models have been proposed highlighting the benefits (Slater et al., 2016). Several studies have developed genomic prediction models for important agricultural traits in potato (Rosyara et al., 2016; Sverrisdóttir et al., 2017; Endelman et al., 2018; Stich and Van Inghelandt, 2018). Chipping quality and starch content prediction models were generated from offspring of 18 diverse breeding varieties (Sverrisdóttir et al., 2017), and total yield, specific gravity, and chip fry color (Endelman et al., 2018) have been developed for potato.

The composition and size of the reference population is critical as only the genetic variation that is present will be predicted, and any that is missing will not have their performance predicted in populations that contain the genetic variants (Riedelsheimer et al., 2013; Stich and Van Inghelandt, 2018). Stich and Van Inghelandt (2018) observed a 10–15% increase in prediction accuracy when predicting with different compositions of a training population. In addition, if there is significant population structure in the reference population that is being evaluated, multiple “population specific” prediction equations may be required. Studies have found clear separation between diploid and tetraploid cultivars (Simko et al., 2006; Stich et al., 2013), and weak sub populations within tetraploids for usage and market release date have been reported (D’hoop et al., 2010). For many potato breeding programs, it is still unclear whether a multi-prediction equation or specific reference populations will be required for each of the market types, and this will depend on the germplasm that each breeding program uses.

Among the various types of markers used, SNP markers are the most abundant in the genome. The generation of the potato genome sequence (The Potato Genome Sequencing Consortium, 2011) revealed the high nucleotide diversity found in potato, estimating a SNP every 24 bp (Uitdewilligen et al., 2013). This also enabled the release of several genomic, genetic, and phenotypic databases with potato as a focus (Hamilton et al., 2011; Felcher et al., 2012; Hirsch et al., 2013). Implementation of genomics in potato improvement is still in the early phases of development. An initial SNP genotyping array was developed (SolCAP 8303 SNP Chip; Hamilton et al., 2011; Felcher et al., 2012), and more recently, this has been extended to a 20K SNP array (Vos et al., 2015). These arrays were based off sequencing data generated from six cultivars (Hamilton et al., 2011) and then expanded with 83 cultivars (Uitdewilligen et al., 2013) that resulted in a large resource of reliable SNPs.

While the SNP arrays provide useful information and are highly accurate in dosage calling and allow many downstream applications, the reducing cost of sequencing is allowing GBS methods to become a viable genotyping option as such systems provide genome-wide SNPs at a lower cost per SNP than SNP arrays (De Donato et al., 2013). However, SNP genotype calling of GBS approaches is typically more intensive and accuracy of dosage will be directly linked to sequencing depth which has cost implications. There are several methods that can be applied for GBS. A complexity reduction approach works by capturing a representable subset of the genome, often through the use of restriction enzymes (Davey et al., 2011). Sequence capture methods are very reliable and easy to optimize, provided a reference genome is available for downstream analysis. More recently, an enhanced restriction-associated DNA sequencing (RAD-seq) approach through optimal enzyme pairing was developed by Jiang et al. (2016). By applying a combination of *Eco*R1 and *Msp*I to reduce chloroplast and rDNA sequences in the libraries, they could call 5,000 variants in 12 potato genotypes. Despite differences in methodology, both GBS and RAD-seq approaches operate by sequencing a set of restriction fragments, typically between 150 and 400 bp (Elshire et al., 2011). Uitdewilligen et al. (2013) used a somewhat different GBS approach by fragmenting and capturing targeted specific DNA using probes from selected genes. Following sequencing of the genomic fragments, almost 130k variants within 807 genes were characterized. The difference with such targeted approaches is that they rely on comprehensive genomic sequence data already existing, as well as known SNP targets to allow the design of the large number of probes. There are often differences in probe binding efficiencies due to regions with a high GC content as well as indels and probe length which can also cause probe failures (Mertes et al., 2011; Uitdewilligen et al., 2013). However, these necessary resources are often available and can be generated with modest effort and investment where required.

The initial development of genomic selection resources benefit from higher levels of SNP coverage across the genome, particularly for traits controlled by a large number of loci, before more targeted genotyping can be designed (Malmberg et al., 2017). Knowledge surrounding blocks of LD and haplotypic structure of the genome enables efficient imputation to be employed to reduce the requirement of such dense SNP genotyping. Genotyping variants in tetraploid species such as potato produces more challenges due to a given gene being represented by up to four different alleles per locus per genotype. When genotyping variants in potato and other highly heterozygous polyploid species, consideration must be given to the genotyping system in use and its ability to distinguish between alleles and quantify the allele number. When provided sufficient read depth is obtained, GBS can provide accurate allele dosage estimates (Malmberg et al., 2017).

For plants where genome size, different levels of ploidy, and frequency of SNPs differ from species to species, a method of complexity reduction for GBS is skim GBS-transcriptomics (GBS-t). GBS-t can enhance sequence alignment by removing complex repeat regions in the genome to focus on the transcriptome, where the size is relatively conserved across species. Other benefits include the low read depth required per genotype for accurate imputation (c. 3 million reads for diploid organisms), even sampling distribution of genes across the genome and that all SNPs identified are found in genic regions (Malmberg et al., 2017). Previous studies have identified the vast majority (>95%) of transcripts that are expressed in leaf tissues (Sudheesh et al., 2016). Futhermore, lower expense through automation of single leaf sampling, lower library costs through reaction miniaturization, and the decreasing cost of sequencing (Malmberg et al., 2017) make this method broadly applicable and high-throughput while still producing a significantly larger number of variants compared to SNP chip assays. With mRNA, splice junctions may cause downstream issues during analysis; however, these issues can be avoided by either aligning to a coding DNA sequence reference or through appropriate selection of analysis software. Previous work (Pembleton et al., 2018) implemented GBS-t on cultivars that are outbreeding populations where pools of plants were genotyped and allelic frequencies were obtained. These frequencies enabled genomic selection for vegetative biomass in perennial ryegrass across multiple seasons.

Recent developments of large genomic, genetic, and phenotypic data sets have assisted with breeding approaches (Hirsch et al., 2014) yet more advances can be made. In this paper, we implement the GBS-t approach on a collection of potato cultivars for marker discovery. The data set was also evaluated in conjunction with historical phenotypic data, to assess the method’s applicability to enable GS across a range of traits and heritabilities. This study aims to assess the GBS-t method and its potential to be used in a GS potato breeding context.

## Materials and Methods

### Plant Materials and Phenotypic Data

The germplasm collection used in this study comprised a total of 181 unique tetraploid potato cultivars. Cultivar names and market class are listed in Supplementary File 1. All phenotypic data associated with traits and characteristics described in this study were obtained from the Australian potato breeding program.

Phenotypic data were collected from trials conducted between 2007 and 2012 at Toolangi, 169 Victoria, Australia. Specific trait analyses for quality traits were conducted at Knoxfield, 170 Victoria, Australia. A minimum of two replicates per trial were used for all cultivars, up to a 171 maximum of 10. All field based and quality phenotypic data were the result of the average of a 172 minimum of 3 years of trialling. An initial analysis of the raw phenotypic data was performed and only phenotypic traits that had the necessary variance and where more than 50% of cultivars had phenotypes were included in the analysis. Flesh color, color when boiled (base flesh color remaining post boiling), skin texture (using an ordinal scale with diseases recorded separately), eye depth, and maturity were scored visually, and crisp score was assessed visually after frying (using USDA chart to normalize year to year), while dry matter was calculated by weighing tubers in air and in water. All the historic phenotypic data, where necessary, was converted from a descriptive to a numerical scale. Conversion of phenotypic data to numerical scales, representation of the variance, and description of numerical phenotypes are provided in Supplementary File 2.

### mRNA Extraction and Sequencing

Tubers from each of the 181 potato cultivars were grown in a pine bark-based potting mix with appropriate nutrients in 6-inch pots under glasshouse conditions. In all cases, the sixth leaf was sampled for an automated 96 well format mRNA extraction. A Dynabead extraction and library preparation method was performed as per Malmberg et al. (2017) including the Sure Select (Agilent) library prep system and their barcodes, modified from Kumar et al. (2012). Library fragment size and quality were analyzed using a TapeStation 2200 platform (Agilent Technologies) (average fragment size c. 275 bp) and pooled and quantified using the Qubit 2.0 Fluorometer (Thermo Fisher). Sequencing data were generated using an Illumina HiSeq 3,000 2 × 150 paired end reads, aiming for 3 million reads per sample. All sequence data are deposited in the SRA database under the BioProject id of SUB4142099.

### Bioinformatic SNP Discovery, Filtering, and Annotation

All data were processed with a single bioinformatic pipeline for SNP discovery. Initial sequence data fastq files were processed through a custom perl script for read quality trimming (minimum read quality score of 20 required) and adaptor removal using cutadapt v1.9 (Martin, 2011). Alignment of the trimmed sequence data to the reference *Solanum tuberosum* Group Phureja DM pseudomolecule (v4.03) assembly (The Potato Genome Sequencing Consortium, 2011; Sharma et al., 2013) was performed using the Spliced Transcripts Alignment to a Reference (STAR) software v2.5.3a using default settings (Dobin and Gingeras, 2015). Using Picard v2.1.0 (http://broadinstitute.github.io/picard) the resulting sam files were cleaned using cleansam for soft-clipping beyond-end-of-reference alignments and setting MAPQ scores to 0 for reads that were unmapped. Files were then converted to bam files with Picard v2.1.0^1^.

Bam files were initially processed with Picard v2.1.0 to mark and remove duplicate reads. The Genome Analysis Toolkit (GATK; McKenna et al., 2010) was used for base-score recalibration and variant calling using the following parameters, all of which needed to be met: quality of mapped read >30; base quality >20; more than five reads covering the base in every genotype; more than four reads covering the alternate base (relative to the reference used) in at least one genotype; and a minimum alternate allele fraction of 0.4. SNP calls (minimum two alternate bases at dp 5 to call heterozygote sample) and genotype assignment (AAAA, AAAB, AABB, ABBB, or BBBB) were done using the HaplotypeCaller function in GATK.

The data were further filtered, and the extraction of all genotypes was processed through R (R Core Team, 2014). Variants were filtered, discarding variants that had 50% or more missing data and removing variants that had a minor allele frequency (MAF) of 5% or less. The resulting SNP data set was annotated using SnpEff (Cingolani et al., 2012). The SnpEff binary database was generated using the whole genome reference sequence and an edited version of the PGSC *Solanum tubersosum* annotation file v4.03 due to overlapping entries.

Missing genotype data were imputed using a custom in-house linkage disequilibrium *k*-nearest neighbor imputation method as described in Pembleton et al. (2018), originally reported in (Money et al., 2015), where genotype classes were converted to reference allele frequency (e.g., AABB = 0.5). Parameters for imputation were 11 nearest neighbors (*k*) and 17 closest loci, and these values were previously found to be highly accurate for more diverse outcrossing populations (Pembleton et al., 2018).

### Data Analysis, Genetic Relationships, and Genomic Selection

Nei’s pairwise genetic distance as a representation of genetic relatedness across the population was calculated using StAMPP (Pembleton et al., 2013). Subsequently, a neighbor joining dendrogram was generated and displayed in DARwin v6.0.5 (Perrier and Jacquemoud-Collet, 2006). SNP distribution and gene density across the genome were assessed by analyzing filtered SNP density and coverage of genes on a 10 KB bin basis across the genome using vcftools (Danecek et al., 2011).

Genomic prediction accuracy was explored with 5-fold cross validation approach where the population was split into five groups. Each group was then genomically predicted using the remaining four groups as the reference population. Genomic predictions were calculated using the BayesA and BayesB models as proposed by Meuwissen et al. (2001) and implemented with the R package BGLR (Perez and de los Campos, 2014).

$$y=u1_{n}+Zv+e$$

where *y* is the trait of interest, *u* is the population trait mean, *Z* is a matrix of genome-wide distributed SNP markers coded as the reference allele frequency. For the BayesA model, $v\sim N\left(0,\sigma_{v}^{2}\right)$ is a vector of random SNP effects estimated from the reference population, and $e\sim N\left(0,\sigma_{e}^{2}\right)$ is a vector of residual errors. While in the BayesB model, $v_{k}=\;\left\{\begin{array}{c} 0\\ \sim N\left(0,\sigma_{v_{k}}^{2}\right) \end{array}\;\begin{array}{c} \mathrm{with}\;\mathrm{probability}\;\pi \\ \mathrm{with}\;\mathrm{probability}\;\left(1-\pi \right) \end{array}\right.$ is a vector of random SNP effects estimated from the reference population with a prior probability *π* that SNP *k* has zero effect. The variance from each SNP, $\sigma_{v}^{2}$ was sampled from an inverted chi-squared distribution using the default degrees of freedom and the scaling parameter determined by BGLR from a trait heritability as listed in Table 2. Marker effects were calculated with 12,000 iterations, discarding the first 1,000 as burn-in. For the BayesB model, the prior probability that the SNP has a non-zero effect (probIn) was set at 0.1, 0.05, 0.01, and 0.005. The proportions were arbitrarily selected, but covered a range of variation that could evaluate the approach potential. The count parameter was set to 10,000. Prediction accuracy was calculated for each *k*-fold as the correlation between the predicted genomic estimated breeding values (GEBVs) and observed phenotypic values. A mean prediction accuracy and standard error were then calculated across the five-fold accuracies. Bias in genomic prediction was also calculated by regressing the observed phenotypic value on the all predicted GEBVs and calculating the slope. A slope coefficient of one was taken as representing no bias, while coefficients greater than one represent underprediction.

To further evaluate marker-trait linkages, the same traits in the above section were evaluated for marker effects. Effects were squared for better discrepancy between markers. Data for estimating marker effects were processed using BGLR, and Manhattan plots were generated in R for eye depth and maturity (Figure 4). Final estimated marker effects were then exported and used to generate Manhattan plots with the R package CMplot (LiLin-Yin, 2018).

## Results

### SNP Discovery and Genetic Diversity in Transcriptome Aligned Data

**Figure 1:**
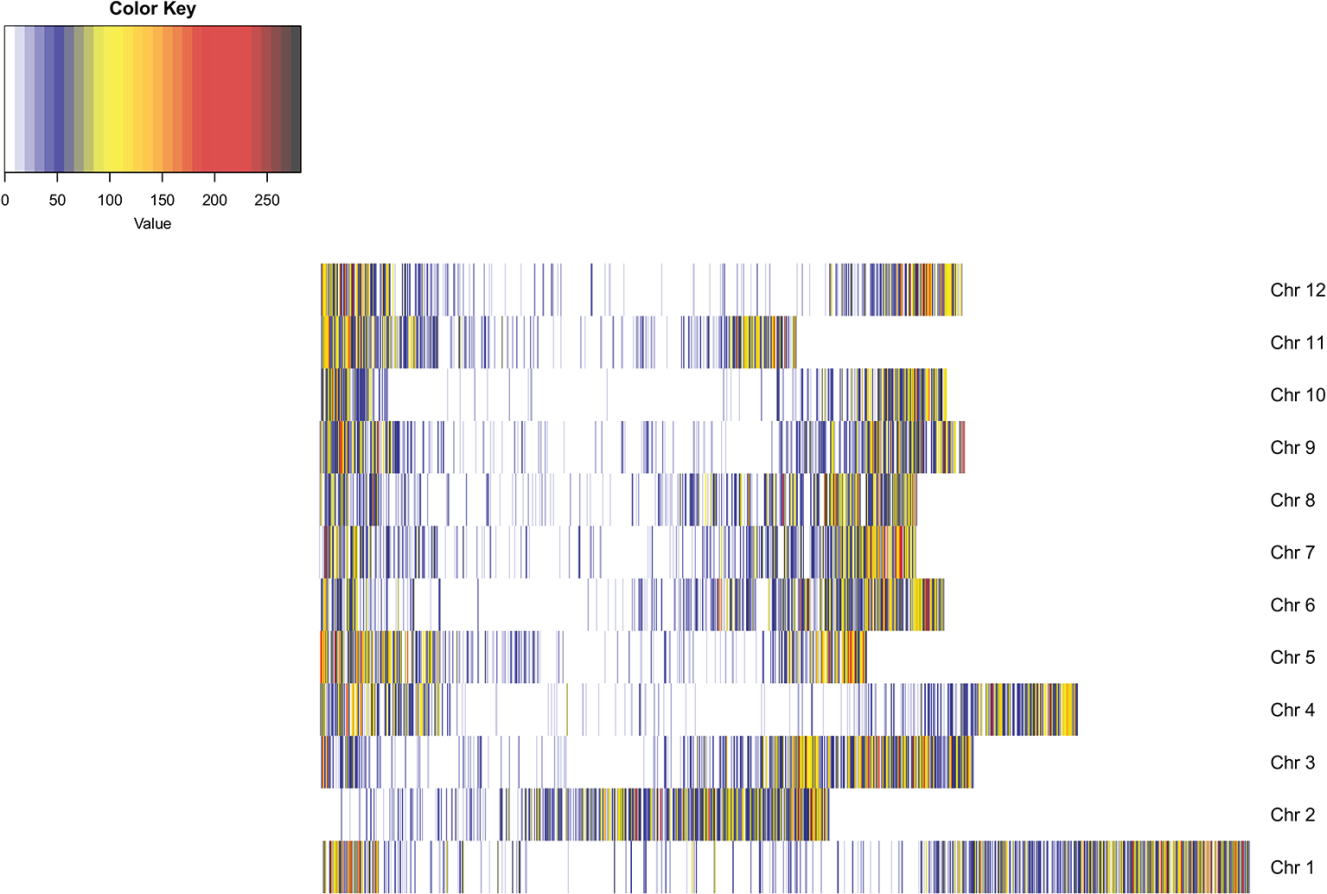
Heatmap of SNP density across the potato genome. SNP density is shown per chromosome in 100 KB blocks. The color scale indicates the density of markers in that segment of the chromosome (blue, low density; red, high density). SNP density is shown to increase toward the telomeric ends of the chromosomes where gene density is higher. Dark blue regions are indicative of centromeric regions.

An average of 3,108,151 reads per sample was generated from the 181 cultivars, with >79% of all cultivars having over 1M reads and over 89% of the high-quality reads aligned to the reference genome. A total of 3,971,538 SNPs were initially discovered, then following the application of stringent read depth, missing data and minor allele frequency filters, 183,848 high confidence SNPs remained (Supplementary File 3). The high confidence SNPs were evenly distributed amongst the chromosomes with at least 10,000 SNPs present on each. However, SNP number per chromosome was not always proportional to chromosome size. This was demonstrated by chromosome 11, the smallest chromosome, which had the third largest number of SNPs (Table 1). Evaluation of genomic distribution of SNPs showed that SNP density is higher toward the telomeric ends of the chromosome and much lower in the regions of predicted centromeres (Figure 1).

**Table 1:** Distribution of filtered SNPs called from RNA-seq data across chromosomes. Gene counts exclude annotations from unaligned contigs in the reference (Chromosome 00).

Chromosome	Length (bp)	Genes per chromosome	Number of SNPs detected	SNPs per gene
1	88,663,952	4,692	13,446	2.9
2	48,614,681	3,214	12,771	4
3	62,290,286	3,601	10,468	2.9
4	72,208,621	3,441	13,921	4
5	52,070,158	2,642	13,437	5.1
6	59,532,096	3,295	14,444	4.4
7	56,760,843	2,711	15,455	5.7
8	56,938,457	2,698	14,963	5.5
9	61,540,751	3,012	15,236	5.1
10	59,756,223	2,847	18,552	6.5
11	45,475,667	2,423	17,689	7.3
12	61,165,649	2,906	23,466	8.1
Total		37,482	183,848	

**Figure 2:**
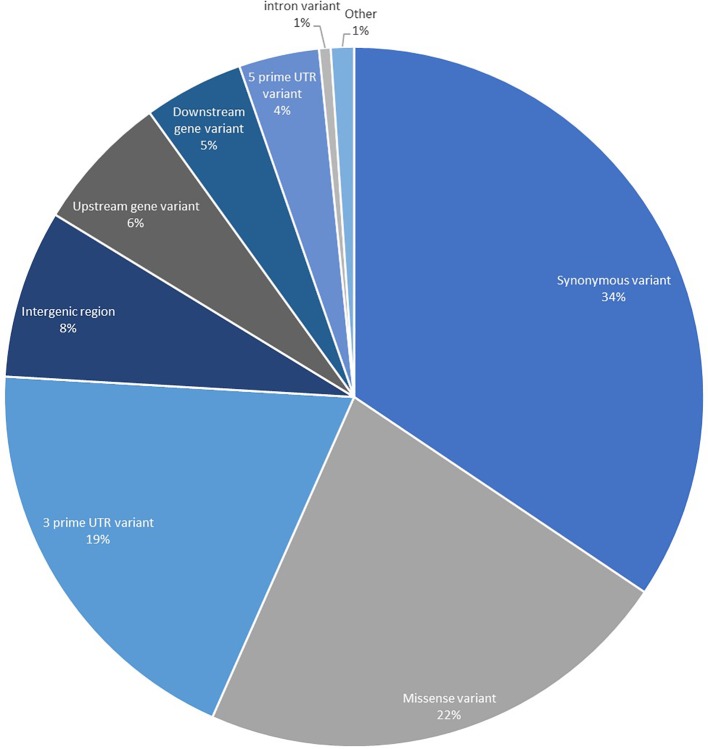
Annotation of 183,848 high-quality transcript SNPs using SnpEff showing the classification of SNPs based on effects. The category titled “Other” is comprised of the classes: non-coding transcript exon variant, splice acceptor variant, splice donor variant, splice region variant, start lost, stop gained, and stop lost.

To obtain genomic variant annotations (defined as assignment of variant function) and ascertain potential functional effects for the complete SNP set identified, all 183,848 high confidence SNPs were annotated using SnpEff to provide the most extensive resource possible (Figure 2). The largest proportion of the SNPs was categorized as synonymous variants (34.42%) with the second largest category being missense variants (22.22%). Variants that were located in UTRs, upstream and downstream regions accounted for 33.98%. A proportion of the SNPs were categorized as intergenic (7.78%), and these are believed to correspond to expressing repeat elements and distant UTRs, as well as potential errors in the initial gene prediction models.

**Figure 3:**
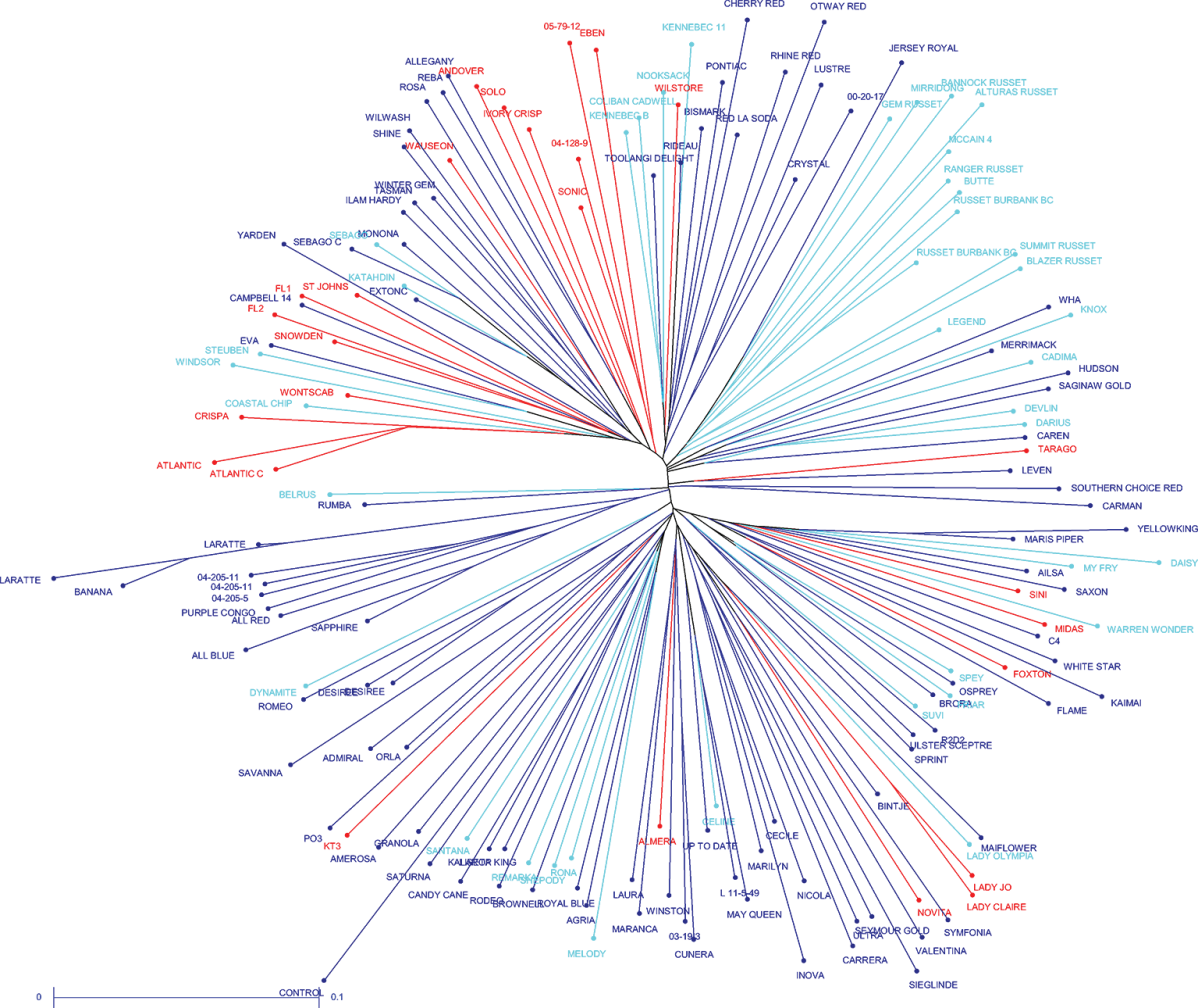
Unrooted neighbor joining dendrogram of the genetic dissimilarity between all samples. Color coding indicates usage type (light blue – French fry; red – crisping; dark blue – fresh).

The potato cultivars that were sequenced more than once and other highly related cultivars showed close relatedness (average distance for complete data 0.3, maximum 0.45, for replicate samples 0.08–0.12). Small clades formed for some usage groups such as French fry, fresh and crisping, for example, the russet samples have a slightly reduced distance (0.26) when compared to the average distance for the complete data. Overall, with the exception of the russet varieties that group together and have the same processing purpose, a lack of population structure is apparent between the 181 potato cultivars, demonstrating broad genetic diversity in the samples chosen. It was also apparent that usage is not a good demarcation of population structure (Figure 3).

### Application of SNP Data Into Genomic Selection

From the complete sample data set, a total of 169 individuals had phenotypic information generated from the historic breeding program activities (Slater, 2016) and were subsequently used for the development of genomic selection prediction equations.

Phenotypic data were available for a range of traits, from highly complex polygenic to known single gene genetic control. Consequently, BayesA and BayesB genomic prediction selection models were explored. BayesA accuracies of 0.81, 0.77, 0.46/0.45, 0.49/0.55, 0.42/0.42, 0.37/0.37, and 0.23 were achieved for flesh color, color when boiled, skin texture, dry matter, eye depth, crisp score, and maturity, respectively (Table 2). For the BayesB models, the analysis was repeated four times with varying levels of markers permitted to be responsible for the trait (Table 2). In the majority of instances, the prediction accuracies for BayesA were inferior to the prediction accuracies that were generated by one of the BayesB thresholds. However, the gains achieved by applying the BayesB approach were minor, for example, the maximum prediction accuracy for flesh color using BayesA was 0.81, while BayesB with a probIn value of 0.01 representing the proportion of markers explaining trait was 0.80 (Table 2). Moderate underprediction bias was observed for eye depth (1.14), crisp score (1.17), color when boiled (1.18), skin texture (1.22), flesh color (1.24), and dry matter (1.39), while minor overprediction was observed for maturity (0.94).

**Table 2:** GS prediction accuracies for seven phenotypes (flesh color, color when boiled, skin texture, dry matter, eye depth, crisp score, and maturity). For each trait, the following is shown: heritability (*h*^2^), maximum theoretical prediction accuracy of the model (Max. potential prediction), accuracy achieved using the BayesA model (BayesA), and the accuracy achieved when using the BayesB model (BayesB) at four settings, differing the probIn at 0.1, 0.05, 0.01, and 0.005.

Trait	*h*^2^^a^	Max. potential prediction^b^	Bayes A	Bayes B			
				0.1	0.05	0.01	0.005
Flesh color	0.8	0.89	0.81 (0.012)	0.80 (0.018)	0.81 (0.019)	0.80 (0.019)	0.81 (0.016)
Color when boiled	0.69	0.83	0.77 (0.014)	0.75 (0.021)	0.72 (0.037)	0.75 (0.022)	0.73 (0.027)
Skin texture	0.5	0.71	0.46 (0.062)	0.50 (0.055)	0.52 (0.054)	0.48 (0.033)	0.56 (0.053)
	0.75	0.87	0.45 (0.064)	0.49 (0.036)	0.51 (0.07)	0.48 (0.029)	0.5 (0.035)
Dry matter	0.5	0.71	0.49 (0.042)	0.48 (0.083)	0.49 (0.075)	0.49 (0.075)	0.54 (0.071)
	0.74	0.86	0.55 (0.071)	0.48 (0.071)	0.47 (0.083)	0.48 (0.072)	0.47 (0.069)
Eye depth	0.5	0.71	0.42 (0.055)	0.44 (0.095)	0.47 (0.091)	0.50 (0.092)	0.54 (0.099)
	0.75	0.87	0.42 (0.055)	0.39 (0.092)	0.39 (0.093)	0.38 (0.094)	0.37 (0.098)
Crisp score	0.59	0.77	0.37 (0.074)	0.46 (0.084)	0.45 (0.078)	0.45 (0.076)	0.46 (0.080)
	0.75	0.87	0.37 (0.078)	0.69 (0.093)	0.69 (0.092)	0.67 (0.089)	0.67 (0.092)
Maturity	0.83	0.91	0.23 (0.061)	0.27 (0.080)	0.29 (0.057)	0.27 (0.067)	0.27 (0.083)

a Heritabilities listed were not calculated from the described phenotypic data set, but are estimates from previous sources (Slater et al., 2014) or have been estimated by correlated traits. Where there are multiple parameters or uncertainty regarding the trait’s heritability, multiple options have been included to allow for different scenarios.

b The maximum potential prediction was calculated by taking the square root of the heritability.

### Estimation of Marker Effects

**Figure 4:**
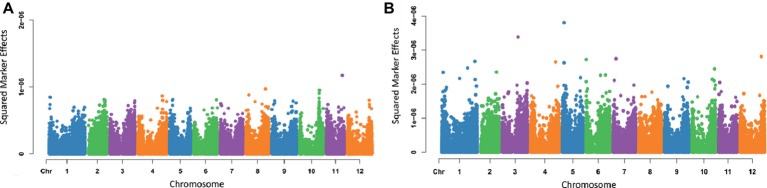
Manhattan plots of squared marker effects estimated for eye depth **(A)** and maturity **(B)** using Bayes A.

Varying magnitudes of marker effects were observed across chromosomes. For the trait eye depth (Figure 4A), the locus having the largest effect was found on chromosome 11; however, most chromosomes had markers that appeared to be contributing to the trait. For maturity (Figure 4B), the largest effect was seen on chromosome 5, but markers varying in effect and contributing to the trait can be seen spread across the entire genome. Marker effect plots for additional traits were generated and are provided in Supplementary File 4.

## Discussion

The difficulties of breeding with autotetraploids are well known but not impossible to overcome. Phenotypic selection has long been the chosen method of breeding for potato when improvement to agronomic and quality traits has been targeted. With the release of the potato genome sequence (The Potato Genome Sequencing Consortium, 2011), reducing sequencing costs and an increase in computational power and analysis tools, the extensive application of GS to potato will become the modern era of breeding. By employing a tailored GBS method for potato, we were able to identify a high number of SNPs in a broadly applicable germplasm collection to enable foundation genomic resources in this area. It should be noted that the computational pipeline used can have dramatic effects on the number and accuracy or SNPs identified and should be designed with as much care and thought as the genotyping assay (Clevenger et al., 2015; Money et al., 2015).

This study confirmed the high sequence diversity of potato previously reported (Hamilton et al., 2011; Uitdewilligen et al., 2013). This approach has been exemplified as effective in both terms of data and cost with large numbers of SNPs being generated from only c. 3 million sequence reads per sample. SNPs were identified in 53,669 genes relating to 95.5% of genes identified in the potato genome, which is in agreement with previous studies (Sudheesh et al., 2016). The overall variant frequency was found to be close to 1 in 20 bp, similar to what was reported by Uitdewilligen et al. (2013) calculated by the number of bases in the transcribed portion of the genome (c. 81Mbp) divided by the complete variant list (c 3.9 M). The highest degree of nucleotide diversity was exhibited on chromosomes 5 and 11. This is unsurprising as chromosomes 5 and 11 contain large clusters of introgressed resistance genes from wild species. In contrast, the lowest diversity was exhibited on chromosome 10. All of these results are in agreement with Uitdewilligen et al. (2013). This can potentially be explained by the large number of conserved genes located near the skin color, tuber shape, and eye depth QTLs that will have been under strong selection in the germplasm evaluated (van Eck et al., 1994, 1995; Li et al., 2005).

The samples used in this study were selected for its diversity and applicability to the potato industries within Australia and globally. There was an apparent lack of population structure within our germplasm, especially when compared to other studies (Hamilton et al., 2011; Uitdewilligen et al., 2013; Boudhrioua et al., 2017). However, by using a higher number of SNP markers and a larger sample number, a more accurate description of the true genetic diversity of the population can be obtained. While acknowledging the value of the previously developed SNP chips (Felcher et al., 2012; Vos et al., 2015), the unavoidable ascertainment bias and cohort of uninformative SNPs reduces the applicability to large data sets (Hirsch et al., 2013). This study illustrates the benefit of using the broadly applicable GBS-t method for SNP discovery on a multi usage collection representative of the major markets, including allele dosage calls at a much lower cost per sample and per SNP than the genotyping arrays. The use of arrays can lead to strong ascertainment bias, especially when an array’s markers were discovered from a small number of samples or samples that do not represent the wider population. General ascertainment bias will always arise from SNP arrays even with a broader discovery panel as genotyping in this manner does not enable ongoing discovery and inclusion of new variants (Albrechtsen et al., 2010; Lachance and Tishkoff, 2013). Additionally, SNP arrays used in potato and other crop species have resulted in large amounts of unusable data (~50%) resulting from missing data, low SNP calling, errors during SNP calling of polyploids, and non-variant SNPs in the populations under investigation. For example, of the 8,303 SNPs on the initial SolCAP array, tetraploid allele dosage frequencies could only be determined with 3,763 SNPs for 250 potato lines (Hirsch et al., 2013) and similarly, for the 20 K SNP array, genotypes were only successfully called for just over 15 K SNPs from a diverse set of 569 potato genotypes (Vos et al., 2015).

The GBS-t approach identifies large numbers of variants that do require strict filtering in order to identify those of high quality. This results in a large amount of unusable data. However, the SNP density in potato is sufficiently high so that less than 5% of SNPS can pass these filters and there are still c. 184 K remaining. This volume of variants is more than sufficient for GS as this study has investigated. An additional issue of the GBS-t method in autotetraploid samples would be accurate allele dosage detection and allele specific gene expression (ASE) causing incorrect genotypes which could have implications on genotype accuracy. In this study, cost effectiveness was a consideration, which limited the sequencing depth performed. As a result, absolute accuracy in genotypes, particularly in the heterozygous class, cannot be evaluated. For this, tetraploids and other polyploids require more sequencing depth which in turn, increases the cost per sample. However, the GBS-t approach was being validated with GS to asses its applicability in routine genotyping in breeding programs. Previous studies have identified that the modest error in genotyping c. 10% has limited effect on GS accuracy (Pérez-Enciso et al., 2015). While the allelic context of potato is more difficult than a standard diploid, the GBS-t method has been successfully applied to determine allelic frequencies in outbreeding perennial ryegrass cultivars across multiple years which is a more complex scenario than is being evaluated in this study (Pembleton et al., 2018).

The genomic prediction accuracy for important agricultural traits across the population was investigated. The highest genomic prediction accuracy was achieved for flesh color and the lowest for maturity. The prediction accuracy for traits typically followed the heritability of the traits (with maturity as an extreme exception). This trend was expected given a higher proportion of the observed phenotype is explained by genetics, rather than environment in those traits with high heritability, such as flesh color. While GS is optimal for highly complex traits, studies have shown that it is still highly effective for traits under simpler control (Daetwyler et al., 2010; Hayes et al., 2010). Prediction bias was observed in the traits evaluated in this study, ranging from minor over-prediction for maturity to moderate underprediction for dry matter. Bias in predictions can have negative effects on selection if plant GEBVs are compared with other plant breeding values that were obtained *via* different methods; however, the method of GS does not typically incorporate such values, and it is proposed that all breeding values be genomically predicted (Lin et al., 2016).

The small population size of 169 cultivars in this study likely contributed to the lower prediction accuracy of those highly complex polygenic traits with lower heritability, such as yield. Traits such as these would require a larger reference population to accurately estimate the individual effects of the large number of loci controlling the trait. This is also supported by the underprediction bias observed for these traits, indicating the genetic effect was not fully estimated, likely due to shrinkage, given the small population size. It is recommended that further studies aimed at developing prediction equations for these traits utilize a much larger reference population. Alternative strategies would be to either include a more diverse selection of cultivars in the population to make a more broadly applicable prediction equation or to focus on restricted specific genetics within specific breeding programs for specific targets. It has been shown that populations with a higher degree of genetic variance have displayed better GS accuracies (Heslot et al., 2012; Crossa et al., 2014). It should also be noted that the material available for this study is advanced cultivars and has already undergone rigorous selection, leading to a reduced spread of some phenotypes. For example, commercial cultivars will have to pass a certain threshold for some common traits in all usage classes, for example, yield and eye depth (71% of all samples with shallow or shallow-medium eye depth). This “bottlenecking” of phenotypes requires less genetic variation for accurate prediction especially when selecting within a population. However, the restricted variance in the population depends upon the heritability of the trait and the proposed degree of variance that is controlling the phenotype. Despite the heritability of these traits and number of markers used in prediction, the lack of genetic variance in this population may have influenced the predictive ability of the models used in this study.

From the evaluation of marker effects, some loci were identified as having larger effects and were concordant with the identified loci previously published. This was shown for traits including maturity and eye depth, where the major locus controlling eye depth has been located on chromosome 10 (Li et al., 2005) and the QTL controlling maturity is located on chromosome 5 (Visker et al., 2003; Gebhardt, 2007; Kloosterman et al., 2013). The region controlling eye depth and an extensive gene list was proposed by Li et al. (2005), and the markers of the highest effect identified in this study fall within this region but in a more refined area. However, a much broader range of effects on other chromosomes was identified (Figure 4A). It is highly likely that the eye depth locus on chromosome 10 has been heavily selected for in all cultivars and therefore does not have the full spread of variation, hence reducing the predicted effect. This shows the benefit of a genome wide association study to understand comprehensively genetic control as well as giving confidence approach taken in this study. The limitation of QTL mapping has historically underrepresented many regions of small effect which have been already identified (Fikere et al., 2018).

In conclusion, this study validates the application of GBS-t as a method for genome wide genotyping in potato with some advantages over other commonly used genotyping techniques. The GBS-t method has also delivered downstream benefits establishing a comprehensive genomic resource of annotated variants for the community. Functional genomic studies on any trait of interest would also benefit from this approach as the resulting data set enables a more complete characterization of the regions of effect, as well as providing gene expression information. It also enables regions of small effect to be identified in a reduced time frame compared to other classical methods. The results will assist in the application of GS in breeding programs for tetraploid potato which enables greater genetic gain per unit time that has been previously described in Slater et al. (2016) as well as a more inclusive understanding of the genetic control of certain traits.

## Author Contributions

BC prepared plant materials, performed RNA extraction, and prepared sequencing libraries. BC, NC, and LP performed data analysis. AS provided the phenotypic data, and BC, FC, BR, AS, and NC conceived the study. All authors read and approved the final manuscript.

### Conflict of Interest Statement

The authors declare that the research was conducted in the absence of any commercial or financial relationships that could be construed as a potential conflict of interest.

## Footnotes

^1^http://broadinstitute.github.io/picard

## Supplementary Material

SUPPLEMENTARY FILE 1 Phenotype data for 169 potato cultivars used in this study with their respective phenotype data.

SUPPLEMENTARY FILE 2 Information on phenotype conversion, phenotypic spread in the population and phenotype descriptions.

SUPPLEMENTARY FILE 3 A variant call format file for the filtered 183,848 filtered SNPs and 164 potato cultivars. This file also contains the SnpEff annotations for each SNP.

SUPPLEMENTARY FILE 4 Marker effect plots for the traits colour when boiled, dry matter, skin texture, crisp score and flesh colour.
