# Supplementary material for: Validation of Genotyping by Sequencing Using Transcriptomics for Diversity and Application of Genomic Selection in Tetraploid Potato
Source: Front Plant Sci. 2019 May 29;10:670. doi: 10.3389/fpls.2019.00670 (PMC6548859; doi:10.3389/fpls.2019.00670)
Supplement: SUPPLEMENTARY FILE 2 — Information on phenotype conversion, phenotypic spread in the population and phenotype descriptions. [file Table_2.DOCX]

Supplementary File 2

Genotyping by Sequencing-Transcriptomics for Diversity and Application of Genomic Selection in Tetraploid Potato

**B.M. Caruana^1,2^, L.W. Pembleton^1^, F. Constable^1^, B. Rodoni^1,2,^ A.T. Slater^1^, N.O.I. Cogan^1,2*^**

**^1^ Agriculture Victoria Research, Agriculture Victoria, AgriBio, the Centre for AgriBioscience, Bundoora, VIC, Australia.**

**^2^ School of Applied Systems Biology, La Trobe University, Bundoora, VIC, Australia.**

*** Correspondence:** Noel Cogan: [noel.cogan@ecodev.vic.gov.au](mailto:noel.cogan@ecodev.vic.gov.au)

# Numerical conversion of phenotypic data

**Table 1.** Conversion of descriptive phenotypes to numerical scales for maturity, flesh colour, eye depth and skin texture

|  | Maturity | Flesh Colour | Eye depth | Skin Texture |
| --- | --- | --- | --- | --- |
| 1 | very very early | white | shallow | smooth |
| 2 | very early | white - cream | shallow - medium | flakey |
| 3 | early | cream | medium | smooth - lightly textured |
| 4 | early to medium | cream - light yellow | medium - deep | lightly textured |
| 5 | medium | cream - yellow | deep | textured |
| 6 | medium - late | light yellow |  | lightly russet |
| 7 | late | yellow |  | russet |
| 8 | very late | dark yellow |  |  |
| 9 | very very late |  |  |  |

# Demonstration of phenotypic spread in the population

**Figure 1.** Phenotypic spread of maturity phenotype in Australian breeding collection

**Figure 2.** Phenotypic spread of flesh colour phenotype in Australian breeding collection

**Figure 3.** Phenotypic spread of skin texture phenotype in Australian breeding collection

**Figure 4.** Phenotypic spread of eye depth phenotype in Australian breeding collection

**Figure 5.** Phenotypic spread of dry matter phenotype in Australian breeding collection

Figure 6. Phenotypic spread of crisp score phenotype in Australian breeding collection

# Definitions of numerical phenotypes

Dry matter: Dry matter was calculated from the specific gravity phenotype. Specific gravity is measured as follows:

SG =     Weight of tubers in air       
      (weight in air) - (weight in water)

SG was assessed through the comparison of the weight of tubers in air compared to their weight in water. Comparison of these values provided an estimation of tuber density, which reflects starch content. From this value, dry matter was calculated using the formula (SG-0.983214)/0.004813.

Crisp Score: Crisp score is a measurement of how well the potato responds to cooking/frying. Slices of potato approximately 1.5 - 2 mm thick were taken from the tubers and cooked in oil, heated to 180 degrees, for 2 minutes. The potato crisps were then scored on the basis of fry colour from 1 (very light) to 10 (very dark) (Slater, 2014). This was normalized from year to year using the USDA chart for French fries and reference photographs for crisps.
