# Supplementary material for: Validation of Genotyping by Sequencing Using Transcriptomics for Diversity and Application of Genomic Selection in Tetraploid Potato
Source: Front Plant Sci. 2019 May 29;10:670. doi: 10.3389/fpls.2019.00670 (PMC6548859; doi:10.3389/fpls.2019.00670)

Supplementary File 4

Genotyping by Sequencing-Transcriptomics for Diversity and Application of Genomic Selection in Tetraploid Potato

**B.M. Caruana^1,2^, L.W. Pembleton^1^, F. Constable^1^, B. Rodoni^1,2,^ A.T. Slater^1^, N.O.I. Cogan^1,2*^**

**^1^ Agriculture Victoria Research, Agriculture Victoria, AgriBio, the Centre for AgriBioscience, Bundoora, VIC, Australia.**

**^2^ School of Applied Systems Biology, La Trobe University, Bundoora, VIC, Australia.**

*** Correspondence:** Noel Cogan: [noel.cogan@ecodev.vic.gov.au](mailto:noel.cogan@ecodev.vic.gov.au)

# Marker effect plots for the traits colour when boiled, dry matter, skin texture, crisp score and flesh colour.


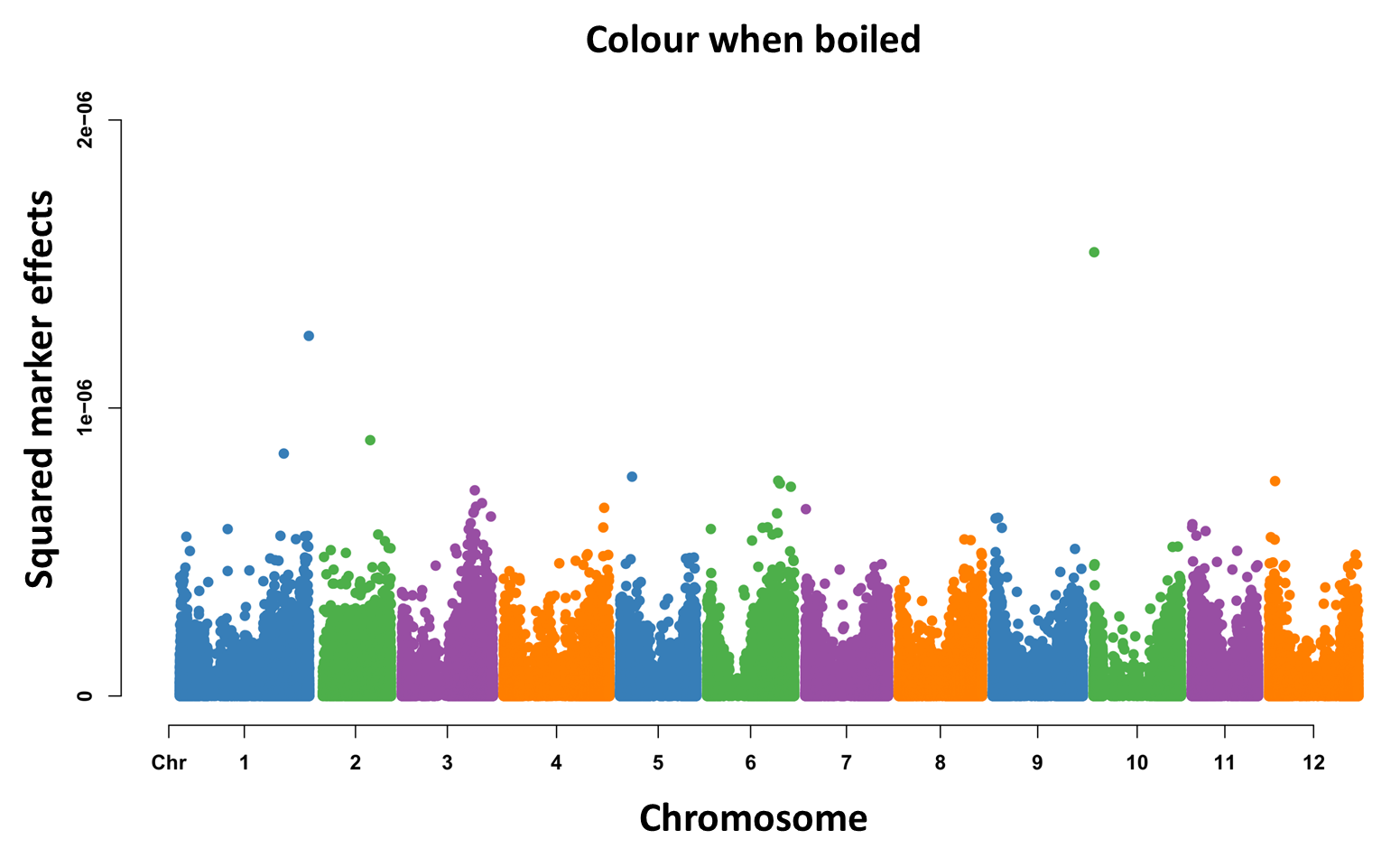


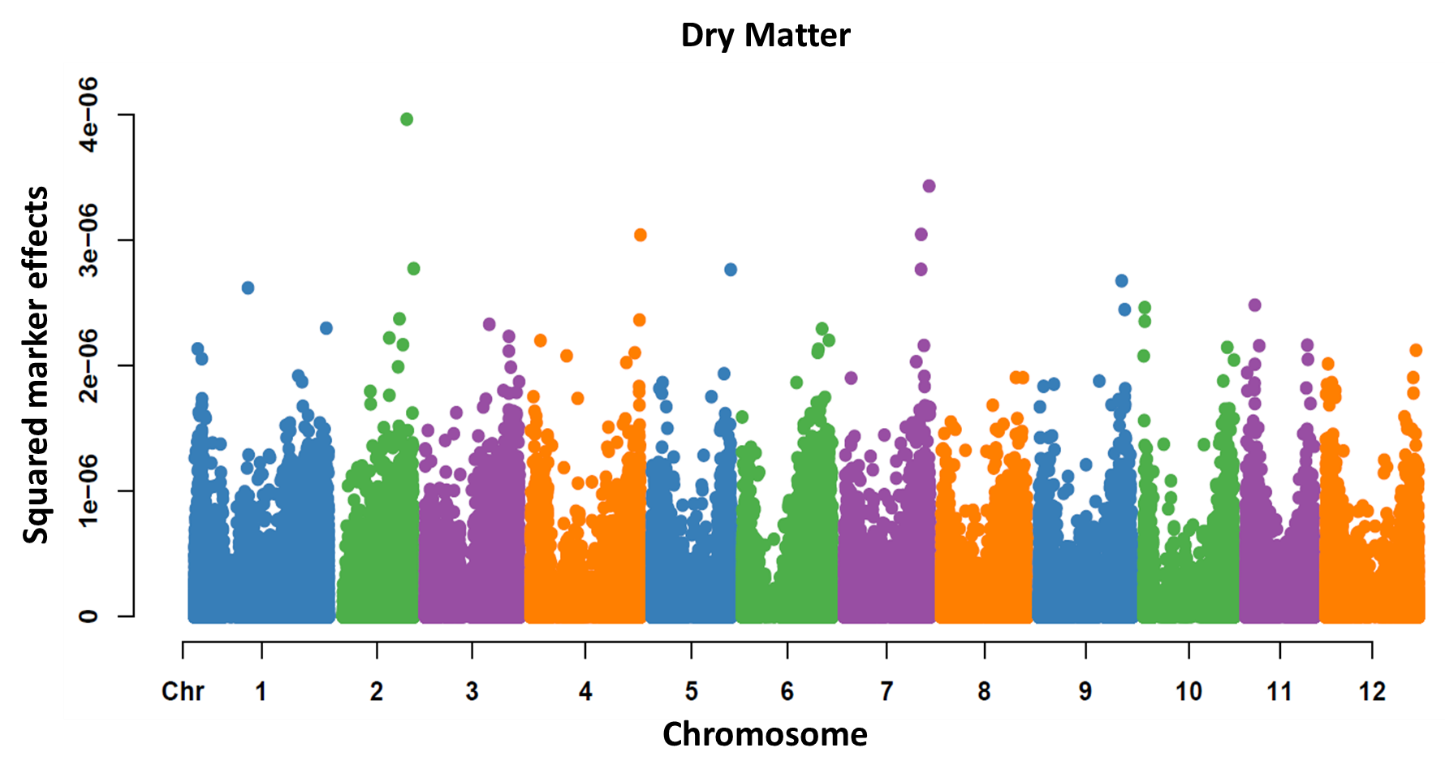


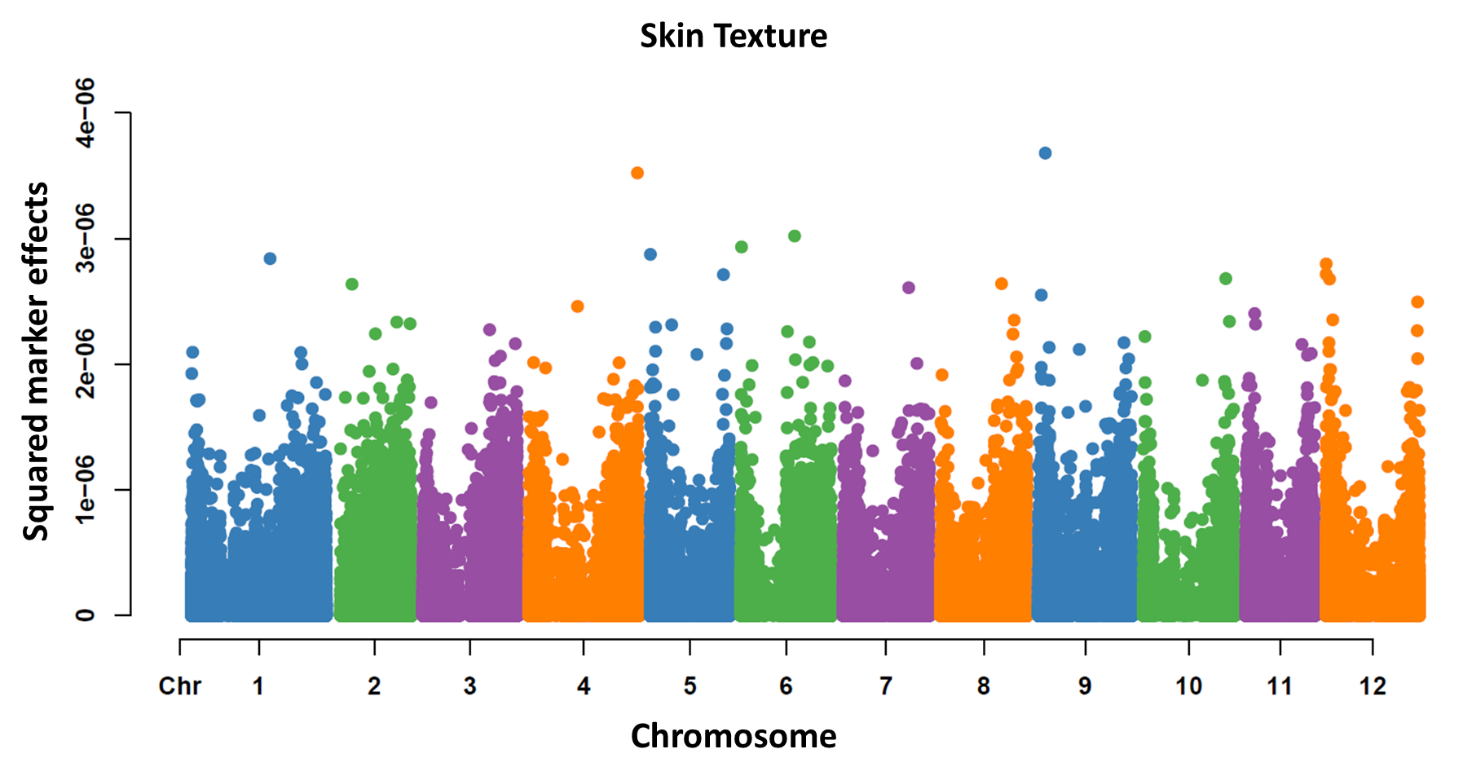


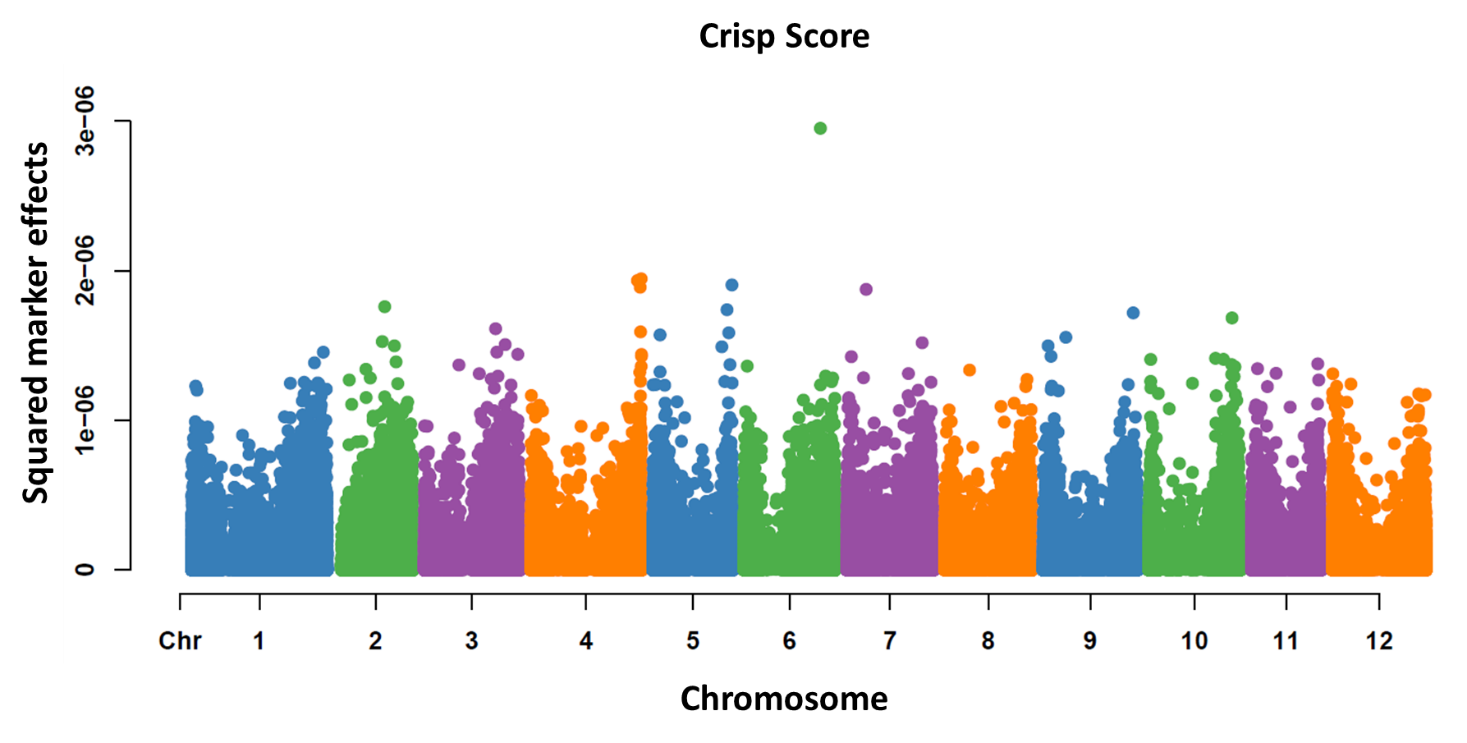


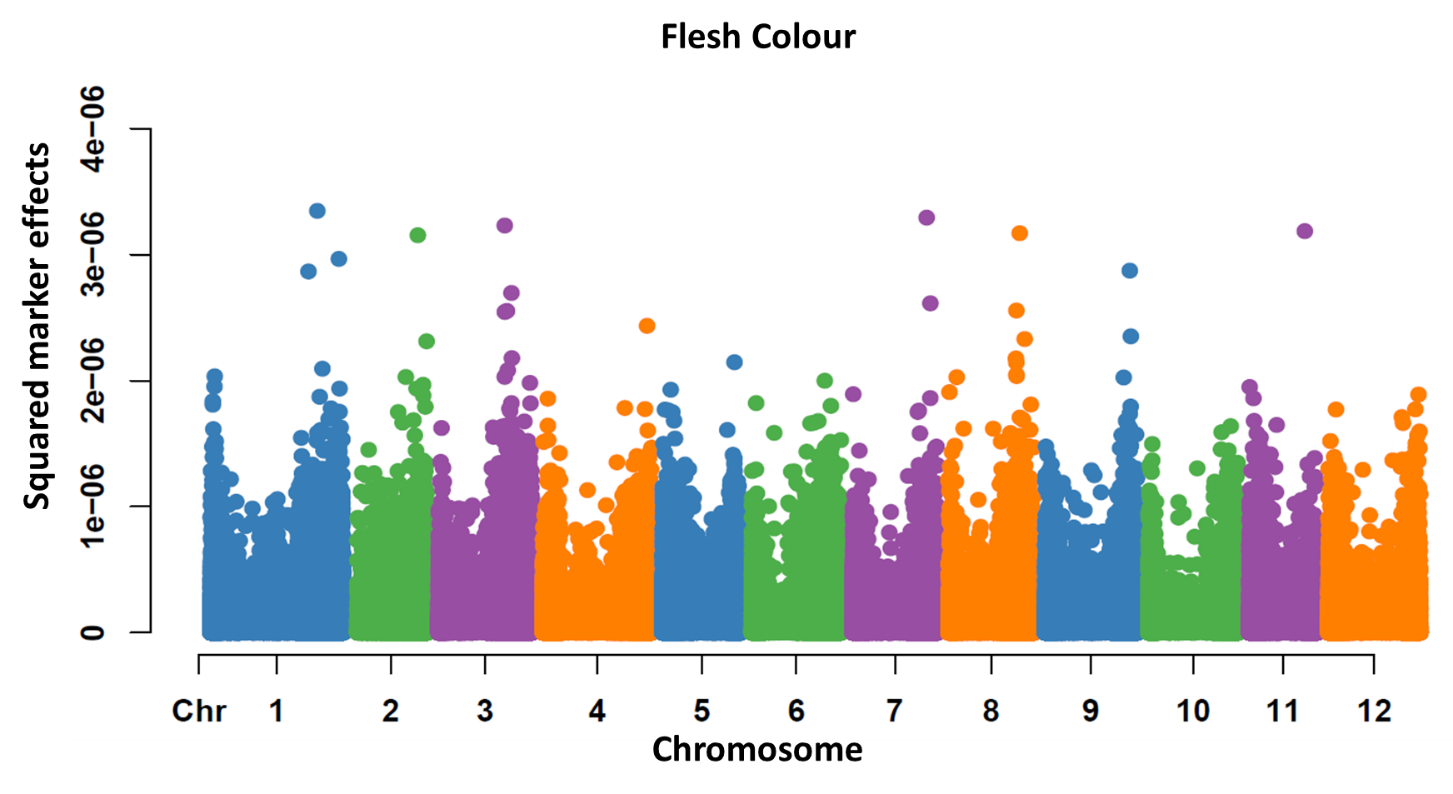

Supplement: SUPPLEMENTARY FILE 4 — Marker effect plots for the traits colour when boiled, dry matter, skin texture, crisp score and flesh colour. [file Table_4.DOCX]
